# Editorial: Modification of polymers with gamma radiation for various high-performance applications

**DOI:** 10.3389/fchem.2022.1042056

**Published:** 2022-09-29

**Authors:** Ajaya Kumar Singh, Rameshwar Adhikari, Md. Abu Bin Hasan Susan

**Affiliations:** ^1^ Department of Chemistry, Government VYTPG Autonomous College Durg, Durg, India; ^2^ School of Chemistry and Physics, Westville Campus, University of KwaZulu-Natal, Durban, South Africa; ^3^ Research Centre for Applied Science and Technology (RECAST) and Central Department of Chemistry, Tribhuvan University, Kathmandu, Nepal; ^4^ Department of Chemistry, University of Dhaka, Dhaka, Bangladesh

**Keywords:** gamma irradiation, polymers, mechanical properties, degradation, cross-linking

Polymer modification by using highly energetic radiations such as gamma rays and the electron beam is a relatively well-established area of research and development. These radiations have been utilized for upgrading the performance of polymeric materials by introducing various architectural modifications and functionalities in the macromolecular chains, desired for particular applications such as in designing and developing biomedical materials, pharmaceutical products, textiles and others. Among various conventional physical and chemical methods, radiation-induced modification of polymers has been more advantageous, due to the ability for rapid generation of radicals in a trunk polymer *via* irradiation without any chemical reagents. The treatments have commonly led to the improvement and/or optimization of various properties such as surface and bulk mechanical properties, electrical and thermal performance, adhesion and processability of the materials. Gamma radiation has been found to be widely applicable in modifying the structure and properties of polymers, and can be used to tailor the performance of either bulk materials or surfaces. The current special issue, comprising five overview articles, has attempted to assemble from leading experts in the field, the recent research developments and applications in gamma-irradiated polymeric materials.

Zhai et al. have presented in detail the results of gamma radiation-modified plastics based on cut-resistant textiles and particularly provided the foundational references on the topic. They have pointed out the importance of the application and modification of high-performance fibers and coating materials in cut-resistant textiles. The thermal degradation and ageing aspects of the gamma radiation-cured polymers have been highlighted by Colin. It has been reported that the mechanical properties of both surface and bulk of the materials get enhanced. The impact of gamma radiation on the degradation behavior of polyesters resins under various solvent environment was explored by González-López et al. They demonstrated that gamma radiolysis of neat resin, in presence some widely used solvents, induced glycosidic scissions on the backbone of the polyester chains. The utilization of the gamma radiations is not limited to the modification of the functional and mechanical properties of the polymers but also supports in the process of protein immobilization on the polymer membranes for their biomedical applications (Schmidt et al.).

The radiation processing has been demonstrated by dozens of studies that the high-energy radiation treatment may have a great impact on optimizing mechanical, thermal, electrical, and surface chemical and mechanical properties and cross-linking.

The gamma radiation-induced chain scission and the resulting grafting and crosslinking are very effective and useful tools in optimizing the mechanical, thermal and electrical properties of polymers (Naikwadi et al.).

The gamma irradiation in controlled fashion was found to enhance the processability of polymers, blends and nanocomposites. The radiation induced grafting of various monomers onto the polymer backbone was emphasized by Naikwada et al. They further performed comparative studies of gamma and electron beams on the mechanical properties of materials. Similarly, comparative studies of gamma and electron beam radiation and their effect on property development have been focused. The high energy radiation modified polymers have been used in several high-performance sectors, including automotive, wire and cable insulation, heat shrinkable tube, sterilization, biomedical, nuclear, and space applications.

Overall, the present collection has offered foundational knowledge on the structure-properties correlations of the gamma rays irradiated polymeric materials and hinted towards the opportunity of more functional modification of the polymeric materials for advanced applications. We take this opportunity to cordially thank all the authors and reviewers for their contribution as well as the *Frontiers in Chemistry* team for permitting us to collaborate in such a highly promising area of applied materials research.

